# Specific Induction of TSLP by the Viral RNA Analogue Poly(I:C) in Primary Epithelial Cells Derived from Nasal Polyps

**DOI:** 10.1371/journal.pone.0152808

**Published:** 2016-04-06

**Authors:** Korneliusz Golebski, Joost van Tongeren, Danielle van Egmond, Esther J. de Groot, Wytske J. Fokkens, Cornelis M. van Drunen

**Affiliations:** Department of Otorhinolaryngology, Academic Medical Center (AMC), University of Amsterdam, Amsterdam, the Netherlands; National Jewish Health, UNITED STATES

## Abstract

**Introduction:**

Chronic rhinosinusitis with nasal polyposis is an inflammatory disease that, although not directly linked to allergy, often displays a Th2-skewed inflammation characterized by elevated local IgE and IL-5 levels. The nasal cavity is constantly exposed to bacteria and viruses that may trigger epithelial inflammatory responses. To gain more insight into mechanisms by which such a biased inflammation might arise, we have investigated the epithelial expression of the Th2 skewing mediators (TSLP, IL-25, and IL-33) in relationship to disease and microbial triggers.

**Methods:**

Epithelial cells were obtained from polyp tissues of nasal polyposis patients and from inferior turbinates of non-diseased controls. Cells were exposed to various TLR-specific triggers to study the effect on mRNA and protein expression level of TSLP, IL-25, and IL-33 and the potential regulatory mechanisms through the expression profile the transcription factors ATF-3, DUSP-1, EGR-1, and NFKB-1.

**Results:**

The TLR3 agonist and viral analogue poly(I:C) induced TSLP mRNA 13.0 ± 3.1 fold (p < 0.05) and protein expression by 12.1 ± 2.3-fold (p < 0.05) higher in epithelium isolated from nasal polyposis patients than in epithelium form healthy controls. This enhanced induction of TSLP may be a consequence of a down-regulated expression of DUSP-1 in polyp epithelium.

**Conclusion:**

The TLR3 induced expression of TSLP introduces a mechanism by which the Th2-skewed tissue environment might arise in nasal polyps and invites a further evaluation of the potential contribution of current or past viral infections to polyposis pathogenesis.

## Introduction

Although the precise pathogenesis of chronic rhinosinusitis with nasal polyps (CRSwNP) still remains unclear, our recent observations have shown a possible important involvement of innate lymphoid cells type 2 (ILC2) in this disease [1]. As differentiation and activation of ILC2 depends on the epithelial mediators TSLP, IL-25, and IL-33 [2], in this manuscript we investigate environmental factors and epithelial regulatory mechanisms that could affect the expression of these key regulatory mediators in primary nasal epithelial cells.

Innate lymphoid cells are relatively new players in the immunological landscape. ILCs share a cellular lineage with T cells, but lack the T cell receptor, precluding any antigen specificity and emphasizing their innate immunological function [3]. Despite this difference, ILCs have defined subtypes that resemble the T helper subtypes with the involvement of the same transcription factors and cytokine profiles. The activity and differentiation of ILC2, the innate equivalent of the type 2 lymphocytes is affected by TSLP, IL-25, and IL-33 in various mouse disease models [1, 4–6]. More importantly, from a human disease perspective is that ILC2 are prevalent in CRSwNP [1, 2] and could therefore potentially explain the overall Th2-skewed local environment prevalent in nasal polyps [7]. Traditionally innate immune responses were linked to adaptive immune responses via dendritic cells with the adaptive Th1 response specific for endosomal pathogens and viruses, the Th2 response for parasite expulsion and allergen-triggered inflammation, and the Th17/Th22 response specific for fungi and bacteria. Specific triggers play an important role in establishing these T helper subtypes. In the case of Th2 responses, a clear role for epithelial TSLP, IL-25, and/or IL-33 have been reported, although different allergic models seem to show differences in the relative contribution of these factors [6, 8–13].

Toll-like receptors are receptors that have been extensively studied in the context of microbiota–triggered local inflammatory responses. These receptors can recognize bacterial derived signals via the plasma membrane associated TLR1, TLR2, TLR4, TLR5, and TLR6, viral components via endosome associated TLR3, TLR7, TLR8, and TLR9, or fungal pathogens via TLR1, TLR2, and TLR4 [14, 15]. Majority of these receptors are expressed by airway epithelium and have been characterized as functional and capable of triggering cell responses upon exposure to ligands [16].

We have previously shown that the ATF-3, EGR-1, DUSP-1, and NFKB-1 transcription factors play an important role in poly(I:C) triggered inflammatory responses and that EGR-1 and DUSP-1 are responsible for a down-regulation of virus-induced inflammation in airway epithelium [17, 18]. In allergic disease we demonstrated that deregulated basal expression levels of the ATF-3, EGR-1, DUSP-1, and NFKB1 genes were associated with differences in cytokine profile of nasal epithelial cells upon stimulation with an exogenous trigger [19]. EGR-1 and DUSP-1 may also play a role in the ILC2 enrichment in CRSwNP as EGR-1 has been shown to mediate the production of TSLP upon IL-33 stimulation [20], while activation of DUSP-1 was necessary for TSLP expression [21].

In this manuscript we explore the reactivity of epithelial cells isolated from nasal polyps and healthy controls to a broad range of TLR agonists in their ability to induce the expression and production of TSLP, IL-25, and IL-33. We also seek to investigate whether EGR-1 and DUSP-1, negative regulators of pro-inflammatory responses, may play a role in regulating these responses. Our data show that epithelial cells from nasal polyps exposed to poly(I:C) produce a significantly higher amount of TSLP than the inferior turbinate epithelium from healthy individuals both at the mRNA and protein level. None of the other TLR agonists (flagellin, PAM, PGN, CpG, or R848) showed a similar effect. For IL-33 we observed a clear induction by multiple TLR agonists, but the level of induction mostly did not differ between healthy or polyp epithelium, although we did see specific up-regulation by the TLR8 agonist R848 in healthy epithelium. IL-25 levels were below the detection limit of our assay for all the conditions we tested. The differences in TSLP expression upon poly(I:C) exposure could potentially be explained by the significantly lower basal expression of the DUSP-1 gene in epithelium from nasal polyposis individuals, since knocking-down of the DUSP-1 gene resulted in a dramatic up-regulation of TSLP expression and production.

## Material and Methods

### Patient Characteristics

Inferior turbinate tissue from six healthy non-diseased individuals was obtained from patients who underwent corrective surgery for turbinate hypertrophy with or without septoplasty. Nasal polyps were obtained from five patients undergoing Endoscopic Sinus Surgery (ESS). None of our subjects had a current respiratory tract infection, none of them suffered from asthma, and none of them was treated with nasal corticosteroids or other nasal medication in the four weeks prior to inclusion. Under Dutch law, tissue removed during mandatory surgery (so not specifically removed for a research purposes) can be used for research when the origin cannot be traced back to the patient. Despite the fact that there is no legal obligation, patients were informed of the intention to use their waste material.

### Cell cultures

Primary nasal epithelial cells were obtained by digesting nasal turbinates or polyps with 0.5 mg/mL collagenase IV (Worthington Biochemical Corp., USA) for 1 hour in Hanks’ balanced salt solution (HBSS; Sigma-Aldrich, NL) followed by an incubation with anti-EpCAM MicroBeads (Miltenyi Biotec, DE) and a positive selection on a magnetic column. Epithelial cells were grown in 75 mL flasks in BEGM growth medium (Lonza Clonetics, NL). Culture medium was replaced every other day. Cells were grown in fully humidified air containing 5% CO_2_ at 37°C.

The human airway epithelial cell line NCI-H292 (ATCC, USA) was cultured in RPMI 1640 medium supplemented with 10% (v/v) fetal calf serum (HyClone, USA), 1.25 mM of glutamine, 100 U/mL of penicillin, and 100 μg/mL of streptomycin. Creation of the EGR-1 and DUSP-1 knock-down mutants is described elsewhere [18].

### Experimental setup

Primary nasal epithelial cells or NCI-H292 cells were cultured to 80% confluence in 12-well plates. 24 hours before the stimulation, culture medium was replaced with serum-free RPMI 1640 medium containing 100 U/mL of penicillin and 100 μg/mL of streptomycin. Cells were then stimulated with the following TLR-agonists: 10 μg/mL PGN (Sigma-Aldrich, DE); 20 μg/mL poly(I:C) (poly(deoxyinosinic-deoxycytidylic acid)) (Sigma-Aldrich, DE); 1 μg/mL flagellin (InvivoGen, USA); 0.5 μM CpG ODN 2216 (InvivoGen, USA) and 5 μg/mL R848 (Sigma-Aldrich, DE). NCI-H292 cells were exposed to 20 μg/mL poly(I:C). Supernatants were removed after 1, 2, 4, and 8 hours of stimulation and cells were used for RNA extraction. For the chemokines/mediators measurements, cells were challenged for 24 hours and supernatants were stored at -20°C until analyzed. Each experiment was performed in biological triplicate.

### RNA extraction and quantitative real-time polymerase chain reaction analysis

Quantitative polymerase chain reaction (qPCR) was used for the determination of the differential expression of selected genes. Extracted RNA (Nucleospin RNA II kit, Machery-Nagel, DE) was used for cDNA synthesis with the MBI Fermentas first strand cDNA kit (Thermo Scientific, NL). cDNA transcripts were quantified by real-time quantitative PCR (iCycler iQ MultiColor Real-Time PCR Detection System; Bio-Rad, FR) with specific primers and IQ^TM^ SYBR Green Supermix (Bio-Rad, FR). The following primers were used for the PCR reactions: GAPDH: 5’-GAAGGTGAAGGTCGGAGTC-3’ and 5’-GAAGATGGTGATGGGATTTC-3; IL-6: 5’- TGACAAACAAAT TCGGTACATCCT-3’ and 5’-AGTGCCTCTTTGCTGCTTTCAC-3’; IL-8: CCACACTGCGCCAACACAGAAATTATTG-3’; 5’-GCCCTCTTCAAAAACTTCTCCA CAACCC-3’; IL-33: 5’-TGTGCTTAGAGAAGCAAGATAC TC-3’ and 5’-GCCTGTCAACA GCAGTCTACTG-3; or with mRNA specific TaqMan gene expression assays (Applied Biosystems, NL) for the following genes: ATF-3 (HS00231069_M1), EGR-1 (HS00152928_M1), DUSP-1 (HS00610257_G1), NFKB-1 (HS00765730_M1), TSLP-total (Hs00263639_m1); TSLP-long form (Hs01572933_m1), and IL-25 (Hs03044841).

Data was analyzed in the Bio-Rad CFX Manager program (Bio-Rad, NL) and fold changes of evaluated genes were calculated using the comparative ΔCt or ΔΔCt method. Each value was corrected for the expression of the housekeeping gene and compared to the control condition. Statistical significance (p < 0.05) was determined by ANOVA and Student’s t-test using SPSS.

### Determination of cytokine and chemokine production by ELISA

Measurements of secreted cytokines were performed by sandwich ELISA in 24-hour cell-free supernatants. The release IL-6, IL-8, IL-33, and TSLP was detected using pairs of specific mAbs and recombinant standards obtained from BioSource International (Camarillo, USA). Statistical significance (p < 0.05) was determined by ANOVA and Student’s *t* test using SPSS.

## Results

### Expression and activity of Toll-like receptors in healthy and polyposis epithelium

**Fig 1:**
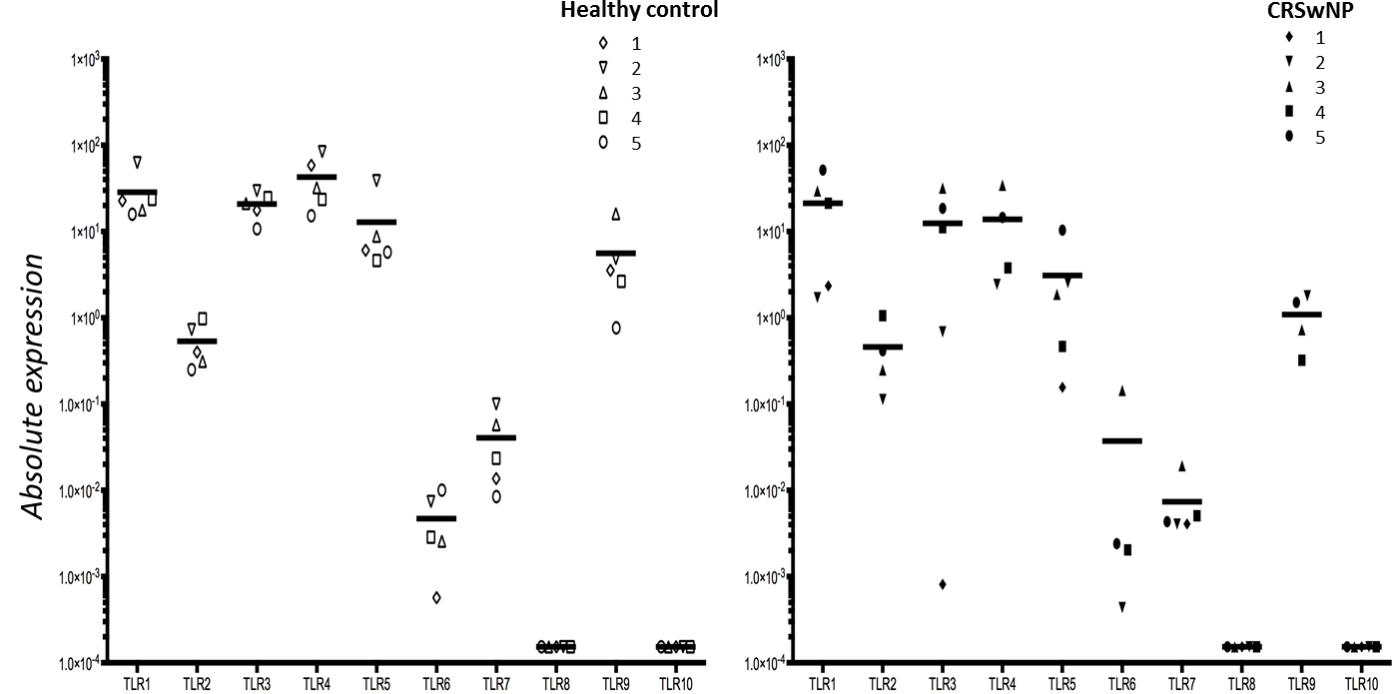
Baseline expression level of TLR1, TLR2, TLR3, TLR4, TLR5, TLR6, TLR7, TLR8, TLR9, and TLR10 in healthy (5 subjects) or polyp epithelium (5 subjects). Expression of each gene was corrected for the expression of the housekeeping gene.

We have previously established the expression profile of TLR receptors in primary nasal epithelium from healthy controls in our experimental model and now we extend this profiling to epithelial cells isolated from nasal polyps. While Fig 1A recapitulates the expression of the various TLRs in healthy epithelium, Fig 1B shows a similar outcome for the TLR expression in nasal polyposis epithelium. Clear expression was observed for TLR1, TLR2, TLR3, TLR4, TLR5, TLR6, TLR7, and TLR9, while we failed to detect TLR8 and TLR10 in nasal epithelium from both healthy controls and nasal polyposis patients. Although we did show expression variations between individuals, none of the TLRs showed clearly significant differences between the two groups. Furthermore, most of these expressed receptors were functionally active given the induced mRNA expression levels of IL-6 and IL-8 of ligation with the appropriate ligand (Tables 1 and 2). Just like previously reported for primary healthy nasal epithelium, also here in epithelium of nasal polyps, despite the presence of TLR4 LPS did not induce IL-6 or IL-8.

**Table 1:** The fold change (FC) induced by different TLR agonists, calculated from the area under the curve for the *IL6* gene expression in a time course over 8 hours in healthy and polyp derived airway epithelium. The value represents an average cumulative gene expression response to the TLR agonists in epithelial cells isolated from 6 healthy controls and 5 polyps with a standard deviation. Statistical significance comparing the differences in cumulative gene expression levels between normal or polyp epithelium is indicated as *p* value.

	Healthy epithelium		Polyp Epithelium		*p* values
	average	st.dev.	average	st.dev.	
**poly(I:C)**	19.72	1.70	17.05	5.40	0.74
**flagellin**	13.05	1.53	9.98	2.58	0.55
**PAM**	15.57	1.70	17.20	3.72	0.55
**PGN**	30.37	7.57	33.87	7.40	0.59
**CpG**	2.38	0.25	2.88	0.32	0.79
**R848**	2.87	0.75	3.20	1.08	0.56

**Table 2:** The fold change (FC) induced by different TLR agonists, calculated from the area under the curve for the *IL8* gene expression in a time course over 8 hours in healthy and polyp derived airway epithelium. The value represents an average cumulative gene expression response to the TLR agonists in epithelial cells isolated from 6 healthy controls and 5 polyps with a standard deviation. Statistical significance comparing the differences in cumulative gene expression levels between normal or polyp epithelium is indicated as *p* value.

	Healthy Epithelium		Polyp Epithelium		*p* values
	average	st.dev.	average	st.dev.	
**poly(I:C)**	9.68	3.57	11.25	9.17	0.88
**flagellin**	6.08	4.52	4.82	3.48	0.39
**PAM**	7.28	7.30	13.27	5.92	0.09
**PGN**	18.55	16.00	27.82	14.63	0.12
**CpG**	1.67	0.47	1.65	0.33	0.92
**R848**	1.72	0.48	1.77	0.27	0.94

### Expression and induction of IL-25, IL-33, and TSLP by bacterial TLR agonists in nasal epithelium

We analysed the impact of microbial stimulation on the potential Th2-skewing ability of nasal epithelium through measuring the basal expression and induction levels of IL-25, IL-33, and the long transcript version of TSLP as functional read-outs (Tables 3 and 4).

**Table 3:** The fold change (FC) induced by different TLR agonists, calculated from the area under the curve for *IL33*) in a time course over 8 hours in healthy and polyp derived airway epithelium. The value represents an average cumulative gene expression to the TLR agonists in epithelial cells isolated from 6 healthy controls and 5 polyps with a standard deviation. Statistical significance comparing the differences in cumulative gene expression levels between normal or polyp epithelium is indicated as *p* value.

	Healthy Epithelium		Polyp Epithelium		*p* values
	average	st.dev.	average	st.dev.	
**poly(I:C)**	3.28	1.22	2.33	0.58	0.12
**flagellin**	2.70	0.80	2.08	0.40	0.67
**PAM**	1.95	0.23	2.08	0.58	0.91
**PGN**	4.12	2.35	3.00	1.25	0.21
**CpG**	2.10	0.17	1.98	0.35	0.88
**R848**	23.18	6.17	1.57	0.38	***0*.*02***

**Table 4:** The fold change (FC) induced by different TLR agonists, calculated from the area under the curve for *TSLP* in a time course over 8 hours in healthy and polyp derived airway epithelium. The value represents an average cumulative gene expression to the TLR agonists in epithelial cells isolated from 6 healthy controls and 5 polyps with a standard deviation. Statistical significance comparing the differences in cumulative gene expression levels between normal or polyp epithelium is indicated as *p* value.

	Healthy Epithelium		Polyp Epithelium		*p* values
	average	st.dev	average	st.dev	
**poly(I:C)**	37.55	7.82	487.80	72.20	***0*.*03***
**flagellin**	227.78	209.42	51.28	55.58	0.12
**PAM**	23.42	31.62	25.00	20.09	0.83
**PGN**	103.30	122.85	165.33	163.28	0.21
**CpG**	3.57	1.45	3.78	2.42	0.88
**R848**	3.32	1.48	6.62	3.22	0.12

No significant differences in the baseline expression of IL-33 between healthy control nasal epithelium or epithelium isolated from nasal polyps could be identified. The induction levels for IL-33 (Table 3) are remarkably similar and range between 2.0 ± 0.2 (p < 0.05) and 4.1 ± 2.35 (p < 0.05) fold for most TLR agonists (flagellin, PAM, CpG, and PGN) acting on epithelial cells isolated from healthy nasal mucosa or nasal polyps. As for TSLP, a moderate induction (3.57 ± 1.45 to 3.78 ± 2.42 fold, p < 0.05) is seen after CpG challenge, while it is much more strongly induced by flagellin (51.3 ± 55.6 and 227.8 ± 209.4 fold, p < 0.05), PAM (23.4 ± 31.6 and 25.0 ± 20.1 fold, p < 0.05), and PGN (103.3 ± 122.8 and 165.3 ± 163.3 fold, p < 0.05) but without any significant differences between healthy epithelium and polyp epithelium (Table 4). In none of the baseline or induced conditions of primary healthy epithelium or epithelium isolated from nasal polyps could we detect any IL-25 mRNA in our real time PCR set up, despite that our protocol was able to amplify IL-25 mRNA from a positive control (data not shown). Although this does not preclude the possibility of very low levels of IL-25 expression we cannot evaluate this mediator in our current experiments.

### Induction and production of IL-25, IL-33, and TSLP by viral TLR agonists in nasal epithelium

To further investigate responses of polyposis epithelium to microbial triggers, we exposed nasal epithelial cells from nasal polyps and healthy controls to viral TLR agonists.

So far, a similar level of induction of IL-6, IL-8, and IL-33 in the epithelia of healthy controls and nasal polyposis patients in responses to different bacterial TLR agonists was shown (Table 1A and 1B and Table 2A) with the viral analogue poly(I:C) (TLR-3 agonist). However, the TLR7/8 agonist R848 triggers the induction of IL-33 in healthy epithelium only (23.2 ± 6.17, p < 0.05) and not nasal polyposis epithelium (1.57 ± 0.38), despite the IL-6 and IL-8 induction levels being similar between those two groups.

The most striking observation is that TSLP expression in nasal polyp epithelium (487.8 ± 72.2 fold, p<0.05) is much more affected by the TLR3 agonist poly(I:C) than in epithelium from healthy individuals (37.5 ± 7.8 fold, p < 0.05), despite no significant differences in IL-6 or IL-8 up-regulation levels between both cell types. Moreover, significant differences, albeit smaller, in the induction levels of TSLP are seen in response to CpG stimulation (3.3 ± 1.48 to 6.6 ± 3.2 fold, p < 0.05).

### Enhanced production and secretion of TSLP in nasal polyp epithelium upon poly(I:C) challenge

Quantification of the TSLP gene level revealed that certain TLR agonists enhance its expression. We sought to validate this observation at the protein level. Epithelial cell stimulated with poly(I:C), flagellin, or PGN produced and secreted TSLP (Table 5). In response to poly(I:C), polyp-derived epithelial cells produce 13.0 ± 3.1 (p < 0.05) fold more TSLP than the healthy epithelial cells. Cell stimulation with flagellin and PGN resulted in modest TSLP secretion, ranging from 7.3 ± 0.9 to 18.3 ± 10.0 pg/mL and showing no statistically significant differences between control and polyposis epithelium. The enhanced production rate of TSLP after poly(I:C) challenge is TSLP-specific, as we could not detect any statistical differences for IL-8 in response to these (and other) triggers between polyp or healthy nasal epithelium (Table 6). Baseline production of TSLP could not be detected in medium collected after 24 hours mock induction. Other TLR agonists did not induce measurable amounts of TSLP despite the significant TSLP-gene expression up-regulation.

**Table 5:** Production of TSLPprotein in cell free supernatants by nasal healthy epithelium or nasal polyp epithelium in response to 24 hours exposure to TLR agonists. Values are shown in pg/mL as average production values from 6 healthy and 5 polyp subjects (b.d. = below detection limit of 1.9 pg/mL). Fold changes (FC) are calculated as a ratio between cytokine production levels in polyposis and healthy tissue.

	Healthy Epithelium		Polyp Epithelium		FC	st.dev.	*p* values
	average	st.dev.	average	st.dev.			
**poly(I:C)**	5.8	0.9	70.2	7.6	12.1	2.3	***0*.*02***
**flagellin**	13.6	3.5	18.3	9.8	1.3	0.8	0.33
**PAM**	b.d.	-	b.d.	-	-	-	-
**PGN**	7.3	0.9	9.6	6.9	1.3	1.0	0.47
**CpG**	b.d.	-	b.d.	-	-	-	-
**R848**	b.d.	-	b.d.	-	-	-	-

**Table 6:** Production of IL-8 protein in cell free supernatants by nasal healthy epithelium or nasal polyp epithelium in response to 24 hours exposure to TLR agonists. Values are shown in pg/mL as average production values from 6 healthy and 5 polyp subjects (b.d. = below detection limit of 1.9 pg/mL). Fold changes (FC) are calculated as a ratio between cytokine production levels in polyposis and healthy tissue.

	Healthy Epithelium		Polyp Epithelium		FC	st.dev.	*p* values
	average	st.dev.	average	st.dev.			
**poly(I:C)**	5008.4	3982.0	4367.3	3433.7	**0.9**	1.0	0.69
**flagellin**	829.4	354.0	1178.7	842.6	**1.4**	1.2	0.19
**PAM**	621.4	120.6	675.4	135.7	**1.1**	0.3	0.81
**PGN**	790.6	174.0	1063.9	280.1	**1.4**	0.5	0.11
**CpG**	49.2	22.4	56.7	29.5	**1.2**	0.8	0.55
**R848**	34.0	24.4	34.5	24.6	**1.0**	1.0	0.91

In the case of IL-33, we could not detect any protein in our ELISA at baseline or after induction of both type of epithelia with any of the TLR agonists, showing that the production rates of IL-33 are at best below the detection limit of the ELISA kit.

### Lower baseline expression of inflammation inhibitors in nasal polyp epithelium

We have previously demonstrated that the ATF-3, EGR-1, DUSP-1, and NFKB1 are affected by the airway epithelium exposure to a virus [17]. Moreover, EGR-1 and DUSP-1 play a critical role in down-regulating the virus-triggered inflammatory responses of epithelium [18]. Therefore, we sought to investigate whether deregulation of the expression of these transcription factors could potentially be responsible for the enhancement of TSLP production in epithelium isolated from CRSwNP tissue.

**Fig 2:**
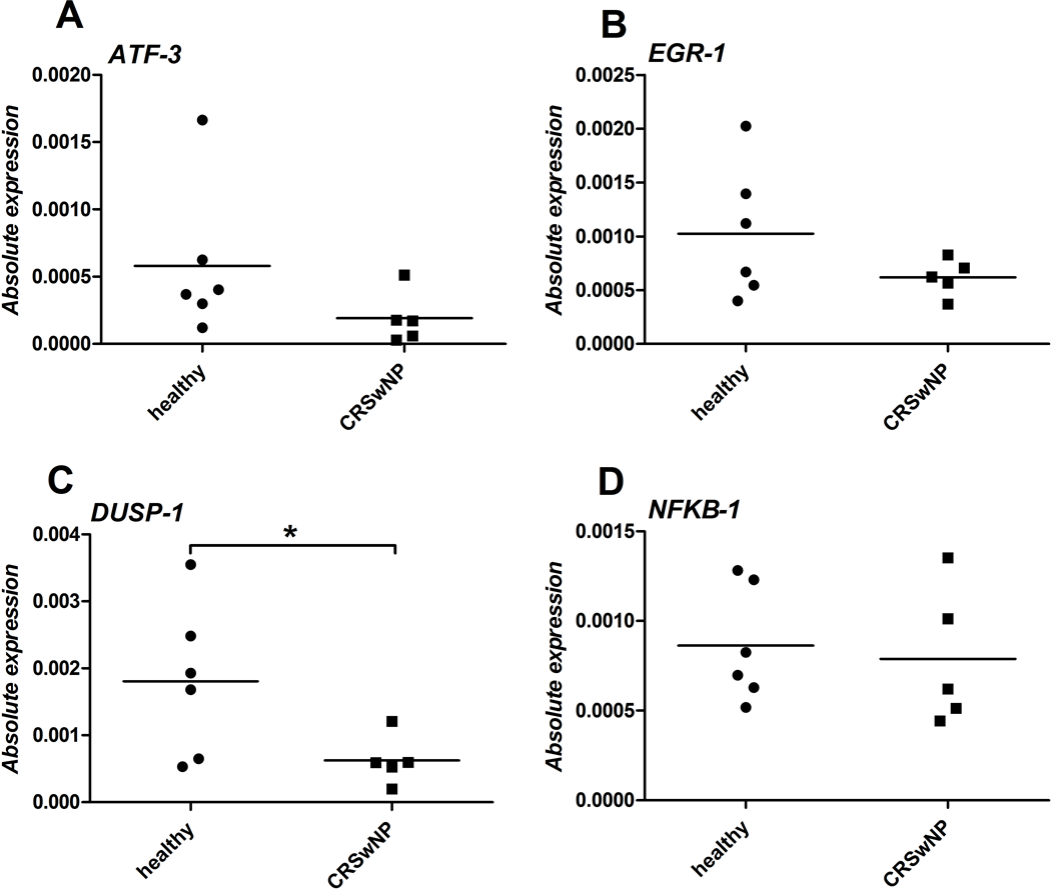
Baseline levels expression comparison of ATF-3, EGR-1, DUSP-1, and NFKB1 in healthy or polyp epithelium. Expression profiles were corrected for the expression of the housekeeping gene. The experiment was performed in a biological triplicate. Statistical significant differences in baseline expression between healthy and polyposis epithelium is indicated (*) if *p* < 0.05.

**Fig 3:**
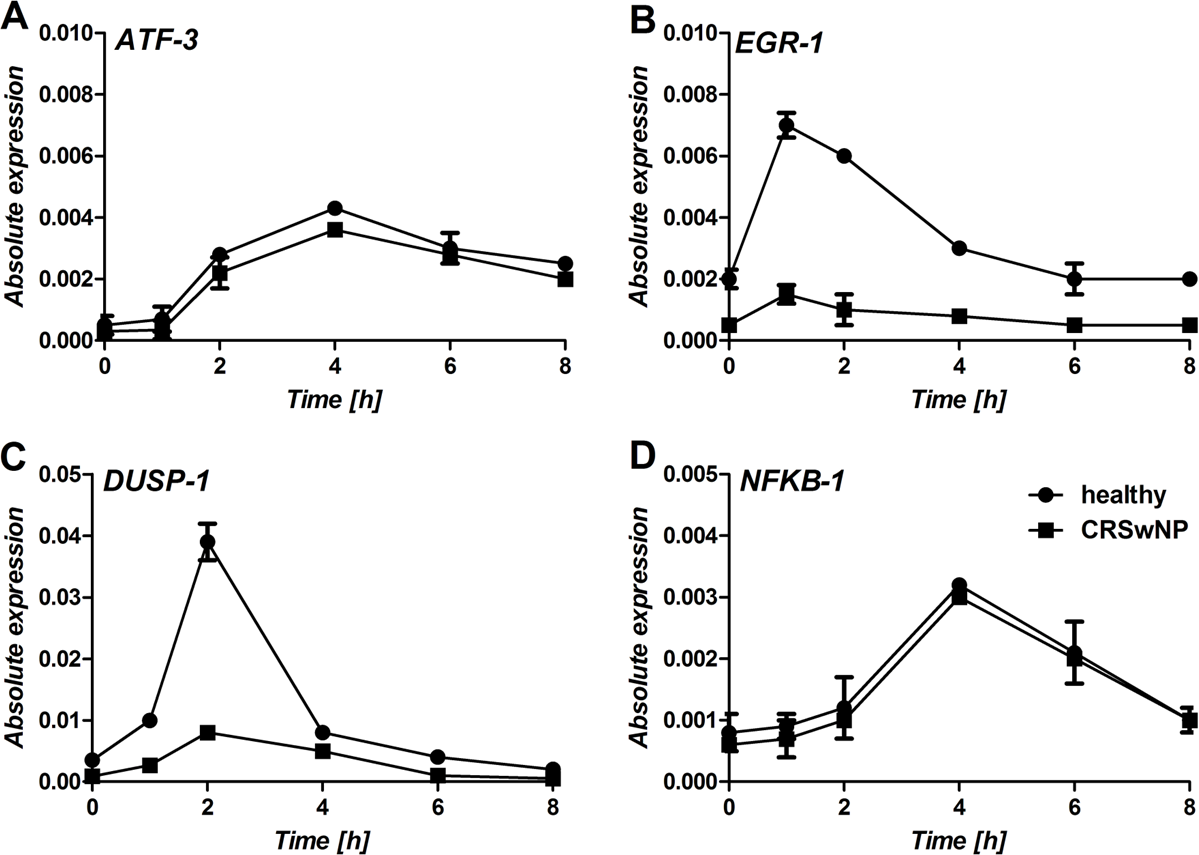
Comparison of expression profiles of A) ATF-3, B) EGR-1, C) DUSP-1, and D) NFKB1 in a time course over eight-hour stimulation with poly(I:C) of healthy or polyposis epithelial cells. Expression profiles were normalized to the control condition with no stimuli and corrected for the expression of the housekeeping gene. The figure shows a result from one representative individual (healthy or CRSwNP) and the experiment was performed in a biological triplicate.

DUSP-1 baseline expression is almost three fold lower (p < 0.05) in polyp-derived epithelium than in healthy epithelium (0.00079 vs. 0.00195). Although not statistically significant, the average basal expression of EGR-1 is almost two fold higher in nasal epithelium obtained from healthy tissue (0.00105 vs. 0.00063, p = 0.065), whereas the average expression of ATF-3 and NFKB1 had similar level for healthy and polyp epithelium (Fig 2). Fig 3 shows ATF-3, EGR-1, DUSP-1, and NFKB1 gene expression profiles in healthy or polyp epithelial cells from two representative subjects in response to poly(I:C). Clear differences in the gene expression profiles of the DUSP-1 and EGR-1 transcription factors between healthy and polyp epithelium are observed. Both in healthy and polyp epithelium, EGR-1 is up-regulated relatively early, reaching its maximal expression level 1 hour after induction in response to poly(I:C), while DUSP-1 reaches its maximal expression level after 2 hours. The area under the expression profiling curves revealed a significant enhancement of up-regulation of EGR-1 and DUSP-1 in healthy control epithelium (2.6 fold higher, p < 0.05 and 1.7 fold, p < 0.05 respectively). ATF-3 and NFKB1 are both induced at the later time points, but none of the transcription factors showed any significant changes in the poly(I:C) timing or induction levels between healthy and polyps epithelium.

### DUSP-1 knock-down results in an enhanced up-regulation of TSLP

**Fig 4:**
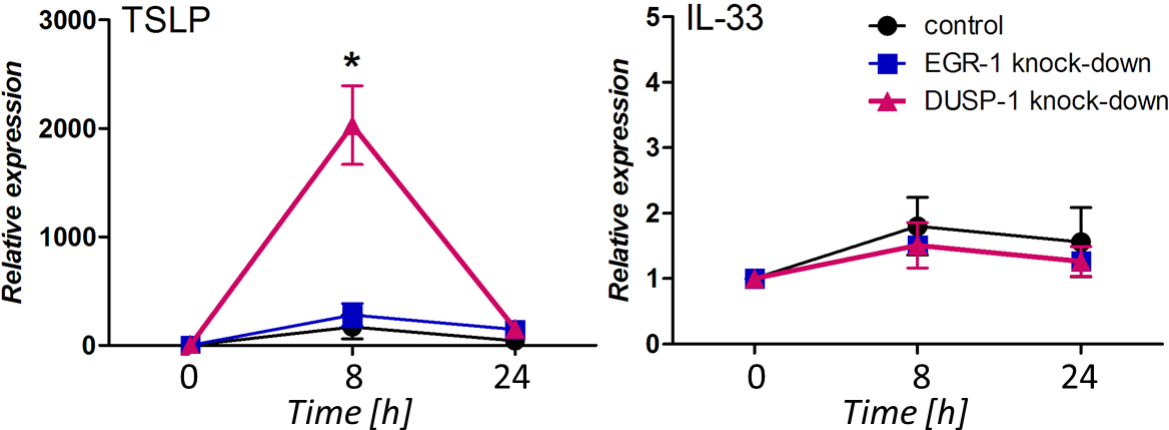
Comparison of expression profiles of A) TSLP and B) IL-33 a time course over 24 hour stimulation with poly(I:C) of NCI-H292 cells. Expression profiles were normalized to the control condition with no stimuli and corrected for the expression of the housekeeping gene. The experiment was performed with three biological replicates on three different occasions.

Previous observations led us to hypothesize that the down-regulated baseline expression of the EGR-1 and/or DUSP-1 transcription factors in nasal polyp epithelium cells may take a part in the enhanced TSLP production upon poly(I:C) exposure. To investigate this in more detail, we performed a targeted EGR-1 or DUSP-1 gene knock-down in NCI-H292 cell that resulted in 92 ± 2% (p < 0.0001) of EGR-1 gene expression reduction and in 76 ± 6% (p < 0.0001) of DUSP-1 reduction compared to expression levels in the control strain. Generated mutant strains were exposed to poly(I:C) in a time course over 24 hours and gene expression and production of TSLP and IL-33 were analyzed. This exposure revealed an enhanced up-regulation of the TSLP gene expression and production in the DUSP-1 knock-down cell line. 8 hours after stimulation, TSLP expression reached 170 ± 101 (p < 0.05) fold up-regulation in the control strain and 280.1 ± 105.5 (p < 0.05) fold up-regulation in the EGR-1 knock-down strain (control vs. EGR-1 KD: p = 0.059), while in the DUSP-1 knock-down strain a strong 2,030.4 ± 362.0 (p < 0.05) and significantly higher than in the control strain (control vs. DUSP-1: p = 0.02) fold TSLP induction (Fig 4A). Upon 24 hours stimulation with poly(I:C), TSLP production was significantly enhanced in the DUSP-1 knock-down cells only, resulting in 45.3 ± 6.3 pg/mL of TSLP release, while in the control and the EGR-1 knock-down strain, TSLP levels were below the detection limit of the kit (2.1 pg/mL). No significant differences in the IL-33 expression or production between the three NCI-H292 strains could be observed (Fig 4B).

## Discussion

In the approach to understand the pathological, often Th2 skewed, mechanisms underlying nasal polyposis, there is a strong emphasis on potential bacteriological contributions and little data to suggest viral involvement. Our data now show that there could be an additional role for viruses that through specific induction of TSLP in nasal polyposis epithelium would be able to contribute to a Th2-skewed environment in polyps.

Nasal polyposis is not strongly linked to clinically defined allergy in the form of allergic rhinitis [7]. However within polyps structures, local presence and production of IgE and prototypical Th2 cytokines can be observed [22–24]. Here we have shown a poly(I:C)-specific induction of TSLP in epithelium from nasal polyposis patients that is much stronger than the induction detected in epithelium from healthy controls. Poly(I:C)-induced expression of TSLP has been previously observed in primary human keratinocytes, conjunctival epithelium, as well as in primary human bronchial epithelial cells [25–27]. The nasal epithelial expression of TSLP does seem to differ significantly from the expression in these other epithelial surfaces. Similarly to keratinocytes, which produce TSLP in response to poly(I:C), flagellin (TLR5 agonist) or diacylated lipoprotein (TLR2/TLR6 agonist) (28), also nasal epithelium secretes measurable amounts of TSLP after TLR3, TLR5, and TLR2/6 triggering.

In this manuscript we touch upon the specificity of TSLP induction in nasal epithelium. At the protein level, we show that upon poly(I:C) challenge increased TSLP secretion is only seen in nasal polyposis epithelium and not in healthy epithelium. Moreover, we see only the increased TSLP induction and no increased induction of the other Th2-skewing mediators IL-25 and IL-33 in nasal polyposis epithelium. The gene expression level brings even more specificity. The increased TSLP mRNA induction was only seen for poly(I:C) and not for any of the other TLR agonists, although at the protein level we could detect a small increase in TSLP after PGN (TLR2/6) and flagellin (TLR5) induction. Although TSLP, IL-25, and IL-33 have all been shown the contribute to the induction of Th2 responses, there seems to be a division of labour where each of the mediators can have a more prominent role depending on the allergy model under investigation [28, 29]. From this perspective we may conclude that TSLP can be a prominent factor in nasal polyposis associated Th2 skewing. However, we do not yet have a clear understanding whether baseline or induced levels of IL-33 could have an additional effect via TSLP or whether TSLP would act independently or in conjunction with other Th2 inducing mechanisms. On the other hand, the induction of TSLP by the viral analogue poly(I:C) is perhaps easier to understand from a mechanistic point of view. At the clinical level there are already many parallels between viral infections and allergy ranging from the induction of allergic exacerbations by viral infections [30] to the slow clearance of virus in allergic individuals [31]. Recently, we have shown that house dust mite allergen- and poly(I:C)-induced responses share a common mechanism [17]. Not only do they induce a similar mediator response, but they also share a common regulatory mechanism through the involvement of identical transcription factors that positively (NFKB-1) or negatively (DUSP-1, EGR-1) affect the epithelial responses seen after allergen or poly(I:C) exposure. Against this light we can understand that a viral analogue can induce the Th2 skewing mediator TSLP. Although in itself the observation that a specific trigger poly(I:C) is able to induce a response in one type of epithelium (diseased polyp) and not in another type (healthy turbinate) seems straight forward, it is particular that this difference in response is seen for TSLP, but not for other outcome measures like IL-6 or IL-8. The 487-fold induction seen for TSLP in nasal polyposis is much stronger that the induction seen for IL-6 and IL-8 in healthy epithelium and furthermore the induction of IL-6 or IL-8 is not affected by the diseased state. This could be partially explained by the relatively low baseline levels of TSLP mRNA, but it does suggest a specific regulatory mechanism for TSLP that is less important for other poly(I:C) induced genes.

We have previously shown that negative regulators of inflammation EGR-1 and DUSP-1 display different levels of specificity depending on what triggers or what outcome measures are measured. In those experiments focusing on inflammatory responses we could show that a knock-down for EGR-1 affected IL-8 expression after house dust mite allergen (HDM) stimulation, but not after poly(I:C) induction, while a knock-down for DUSP-1 affected IL-6 and IL-8 after HDM or poly(I:C) stimulation [18]. In the present study, we show that the baseline DUSP-1 levels are lower in polyp epithelium than in healthy control group and that this may contribute to reduced polyp epithelial cell capability of tuning the viral-induced pro-Th2 inflammatory responses down. Indeed, knocking-down of the DUSP-1 gene in the airway epithelial cell line resulted in a dramatic up-regulation of TSLP after cell exposure to poly(I:C). Interestingly, the level of TSLP enhancement between the control and the DUSP-1 knock-down strain was comparable to that between nasal polyp and healthy control epithelium. Involvement of DUSP-1 in CRSwNP pathology does not come as a surprise since we have previously demonstrated the critical role of DUSP-1 in abating the inflammatory responses upon dexamethasone challenge [18]in airway epithelium. Intranasal administration of glucocorticosteroid is the first line treatment for CRSwNP[7]. Since glucocorticoid challenge enhances the DUSP-1 gene expression and reduces production of inflammatory mediators [18]it may perhaps explain the improvement of the quality of life scores for most CRSwNP individuals, especially those with low baseline expression of DUSP-1.

In conclusion, we have shown that poly(I:C)-induces expression of TSLP in primary nasal epithelial cells from polyposis patients only and that a deregulation of the expression of the DUSP-1 transcription factor may play a role in this process. No other TLR agonists nor primary healthy epithelial cells show the induction of this Th2 skewing mediator. These observations opens the way to explore the involvement of viral infections as a causative or contributing factor to the Th2-skewed inflammatory environment in polyps or to the pathogenesis of nasal polyposis.
